# Symptom clusters and core symptoms in patients with colorectal cancer after postoperative chemotherapy: A cross-sectional network analysis in a predominantly stage II to III cohort

**DOI:** 10.1097/MD.0000000000047647

**Published:** 2026-02-13

**Authors:** Bingbing Xiao, Jiayi Wan, Wendan Jing, Jing Zhao, Tingting Tan, Qijun Lv, Hongyan Kou

**Affiliations:** a Gastrointestinal Surgery 1, Affiliated Hospital of North Sichuan Medical College, Nanchong, Sichuan, China.

**Keywords:** chemotherapy, colorectal cancer, core symptoms, network analysis, symptom clusters

## Abstract

This study aims to investigate symptom characteristics in postoperative chemotherapy patients with colorectal cancer (CRC) and identify their symptom clusters and core symptoms to provide a basis for developing precise symptom management strategies. Convenience sampling was used to select 302 patients with postoperative chemotherapy for CRC as the research object. The general data questionnaire and the Chinese version of the MD Anderson Symptom Inventory–Gastrointestinal Cancer were used for investigation. Exploratory factor analysis was conducted to extract symptom clusters, followed by constructing a symptom network model using R software. Core symptoms were identified by calculating centrality indicators. Exploratory factor analysis identified 5 symptom clusters with a cumulative variance contribution rate of 64.53%, including chemotherapy-related symptoms, psychological symptoms, neurotoxicity-related symptoms, gastrointestinal symptoms, and CRC-specific symptoms. Analysis of symptom prevalence revealed poor appetite (85.4%) as the most common symptom. Network analysis showed that distress (*r*_s_ = 1.04, *r*_e_ = 1.04) exhibited the highest node strength and expected influence, while fatigue (*r*_c_ = 0.0047) demonstrated the strongest centrality, confirming it as a core symptom. Distress and fatigue are core symptoms in the symptom network of postoperative chemotherapy patients with CRC. Clinical interventions should focus on these core targets to alleviate overall symptom burden by blocking interactions between symptoms.

## 1. Introduction

Colorectal cancer (CRC) is one of the most common malignant tumors in the digestive tract. According to the latest data from 2022,^[1]^ CRC accounts for 9.6% of all global cancers, ranking third worldwide. The standard treatment for CRC involves a comprehensive approach with surgery as the primary modality, supplemented by radiotherapy and chemotherapy. While some patients receive preoperative radiotherapy to shrink the tumor, downstage the disease, and improve resectability, most patients start adjuvant chemotherapy around 4 weeks postoperatively to consolidate efficacy and prevent recurrence.^[2]^ Under the dual stress of surgery and chemotherapy, patients often suffer from multiple physical and psychological symptoms such as pain and fatigue, severely compromising their quality of life.^[3,4]^ Network analysis can collectively represent disease-related symptoms, visually presenting complex relationships between different symptoms and offering new approaches to explore their interaction mechanisms. A symptom network refers to a graph formed by all symptoms exhibited by patients at the same measurement time point.^[5]^ High co-occurrence/strongly correlated symptom subsets within the network are termed “symptom clusters,” which consist of 2 or more interconnected yet distinct symptom sets. Identifying core symptoms – key nodes with the highest centrality in the network – through intervention can significantly improve symptom management efficiency.^[6]^

Symptom cluster research in China started relatively late. Current studies primarily focus on symptom cluster extraction in postoperative chemotherapy patients with CRC. Many researchers predominantly use the Anderson Symptom Checklist to assess patients who received their first or at least 1 chemotherapy session after surgery. However, due to the gradual changes in symptom occurrence and severity over time,^[7]^ coupled with variations in analytical tools and statistical methods, there are significant inconsistencies in the number of extracted symptom clusters and the symptoms within each cluster.^[8,9]^ Moreover, existing research has paid little attention to identifying core symptoms within the symptom network. Although some studies have identified common symptoms such as low mood, distress, fatigue, and diarrhea,^[10]^ they have not employed network analysis to clarify these symptoms’ centrality or their core role within the overall network, leading to inconsistent conclusions.

In conclusion, to more precisely improve the quality of life for chemotherapy patients after CRC surgery, this study investigates symptom patterns in CRC patients 2 to 3 days postchemotherapy, when symptoms stabilize. By constructing a symptom network, identifying symptom clusters, and exploring core symptoms within the network, we aim to provide a reference for developing precision-based symptom cluster management strategies tailored to key symptoms.

## 2. Objects and methods

### 2.1. Ethical statement and patient consent

This study was approved by the Ethics Committee of the Affiliated Hospital of North Sichuan Medical College (Approval No. 2025ER73-1). Consent was obtained from all respondents before the study commenced.

### 2.2. Study objectives and endpoints

The primary endpoint of this study was to identify the core symptoms with the highest strength centrality within the symptom network of postoperative CRC patients undergoing chemotherapy using network analysis. The secondary endpoints included extracting symptom clusters via exploratory factor analysis and assessing the prevalence and severity of individual symptoms.

### 2.3. Research object

Due to the relative dispersion of the target population in clinical practice, convenience sampling was the most feasible strategy within the real-world medical setting. This approach ensured efficient data collection within limited time and resources, while also facilitating the rapid acquisition of a sufficient sample size for symptom network analysis. Therefore, this study employed a convenience sampling method to consecutively recruit patients who received postoperative chemotherapy for CRC at the oncology department, gastrointestinal surgery department, and daytime chemotherapy center of 2 tertiary hospitals in the city between January and April 2025.

The inclusion criteria were as follows: diagnosis of CRC confirmed by colonoscopy,^[2]^ having undergone radical resection for CRC and completion of at least one cycle of chemotherapy (predominantly patients with stage II–III disease), age ≥18 years, conscious and providing informed consent, and ability to complete the questionnaire independently or capability to complete it successfully with the assistance of an investigator. The exclusion criteria were as follows: history of other malignant tumors or severe psychiatric disorders, tumor recurrence or presence of multiple distant metastases, and poor physical condition precluding participation in the survey.

#### 2.3.1. Sample size calculation

The MD Anderson Symptom Inventory–Gastrointestinal Cancer module includes 18 symptoms. The total parameters required for constructing a network model are 171 (i.e., 18 threshold parameters and 18 × [18 − 1]/2 = 153 pairwise association parameters).^[11]^ To ensure model reliability, the sample size must match the parameter quantity with a 20% dropout rate, ultimately requiring at least 214 participants.

### 2.4. Survey tools

#### 2.4.1. General information survey form

Developed by the researcher based on similar studies,^[11,12]^ this questionnaire includes demographic data (gender, age, occupation, current residence, marital status, educational background, health insurance type, average monthly income, smoking history, and alcohol consumption history) and medical records (disease location, tissue type, tumor stage, diagnosis duration, preoperative chemotherapy, number of chemotherapy sessions, chemotherapy regimen, radiotherapy status, targeted therapy status, immunotherapy status, stoma presence, distant metastasis status, and number of comorbid chronic diseases).

#### 2.4.2. *MD Anderson* S*ymptom Inventory–Gastrointestinal Cancer*

Developed by the Anderson Cancer Center in 2000^[13]^ and localized by Wang et al in 2004,^[14]^ it primarily evaluates symptom severity over the past 24 hours. The inventory comprises 2 components: a primary section with 13 core symptoms and 5 gastrointestinal tumor-specific symptoms (totaling 18 items), and a secondary section assessing symptom interference with 6 daily living activities. All items use a 0 to 10 numerical rating scale, where 0 indicates no symptoms and 10 represents maximum severity. Both components demonstrate excellent reliability with Cronbach α coefficients of 0.84 and 0.90, respectively. In this study, we conducted symptom network analysis using the primary section’s symptom severity scale, achieving a Cronbach α coefficient of 0.783.

### 2.5. Data collection methods

After obtaining departmental approval, the research team distributed questionnaires 2 to 3 days after the completion of the participants’ chemotherapy. Researchers personally conducted one-on-one interviews with eligible patients, using standardized instructions to explain the study purpose and questionnaire completion methods. Participants independently completed the questionnaires, which were collected on-site immediately after verification. For patients who had been discharged and could not complete the questionnaire in person, we conducted telephone follow-ups where they filled it out through a question-and-answer format. All returned questionnaires underwent rigorous quality checks. The exclusion criteria were questionnaires with over 20% of key data missing and questionnaires exhibiting a fixed response pattern. Specifically, 8 questionnaires were excluded due to identical severity ratings across more than 10 consecutive symptom items (e.g., all “0” or all “10”). This pattern was deemed unlikely to represent a genuine symptom experience and potentially indicated respondent fatigue, difficulty in understanding, or careless responding. Retaining these invalid responses would introduce significant noise and bias the subsequent network and factor analyses. A total of 310 questionnaires were distributed in this study. After excluding 8 valid responses due to regular answering patterns or identical options, 302 valid questionnaires were collected, achieving an effective response rate of 97.4%.

### 2.6. Statistical methods

#### 2.6.1. Statistical description

Descriptive analysis was performed using SPSS 27.0 (IBM Corp., Armonk). For quantitative data, mean ± standard deviation was used when a normal distribution was observed, while *M (P*_25_, *P*_75_) was employed for non-normal distributions. Categorical data were presented as frequency and percentage. Symptom severity scores, which did not follow a normal distribution, were also expressed using *M (P*_25_, *P*_75_).

#### 2.6.2. Cluster analysis

Cluster analysis categorizes multiple symptoms into distinct clusters (symptom clusters) based on symptom similarity (e.g., prevalence, severity). In this study, exploratory factor analysis – which groups strongly correlated symptoms into the same factor to achieve dimensionality reduction and symptom clustering – was applied in combination with varimax rotation. Symptoms with a prevalence >20% were selected for symptom cluster extraction.^[15]^ Before factor analysis, the Kaiser–Meyer–Olkin (KMO) test and Bartlett sphericity test were carried out. KMO value >0.5 and a significant Bartlett test of sphericity (*P* < .05) were considered indicative of data suitability for factor analysis. The selection principles of factors include eigenvalue ≥1, at least 2 symptoms, and symptom load ≥0.4. If the load of the same symptom in multiple factors is all ≥0.4, its belonging is determined according to the maximum load.

#### 2.6.3. Network analysis

Network analysis represents symptoms as nodes in a network. The connections between nodes are referred to as edges, with the thickness of each edge reflecting the strength of the association between nodes. This method is used to identify core symptoms (key nodes) and the strength of symptom–symptom interactions. In this study, symptom networks were constructed using the qgraph package in R version 4.4.3 (R Foundation for Statistical Computing, Vienna, Austria), based on the Gaussian Graphical Model and the EBICglasso algorithm. Unlike symptom prevalence, a core symptom is a key node that plays an important role in connecting and influencing the entire network. Core symptoms can be evaluated by centrality indices; a higher centrality value indicates a more central role within the network. Key centrality indicators include *strength* (the absolute sum of all edges connected to a node), *closeness* (the reciprocal of shortest path lengths between nodes), and *betweenness* (the frequency of a node acting as an intermediary between others).^[16]^ The expected influence provides a comprehensive measure of a node’s stability impact through its connections. Higher expected influence values correspond to greater centrality, indicating increased importance.^[17]^

Previous research^[18]^ indicates that betweenness and closeness centrality are generally unstable. Therefore, this study focuses on node strength and expected influence coefficient. The bootnet package was used to evaluate the stability and accuracy of network structures.^[19]^ Network stability is assessed using the correlation stability coefficient, which should typically be at least 0.25 (ideally above 0.5). A narrower 95% confidence interval for edge weights indicates more accurate estimation.^[20]^

## 3. Results

### 3.1. General data characteristics of patients with postoperative chemotherapy for CRC

A total of 302 patients completed the study. The majority (288 cases, 95.4%) were aged 40 to 80 years, with males constituting the majority (178 cases, 58.9%). The population was predominantly married individuals (278 cases, 92.1%), and most had educational backgrounds equivalent to junior high school or below (244 cases, 80.8%). Farmers and workers accounted for a significant proportion (163 cases, 54.0%). Of the patients, 145 (48.0%) had undergone ≤2 cycles of chemotherapy, 80 (26.5%) had received 3 to 5 cycles, and 77 (25.5%) had completed ≥6 cycles, indicating a relatively even distribution across treatment exposure categories. Other general data characteristics are shown in Table 1.

**Table 1:** Demographic and clinical characteristics of patients with colorectal cancer (n = 302).

Variables	Classify	n (%)
Sex	Male	178 (58.9)
	Female	124 (41.1)
Age (yr)	≤40	12 (4.0)
	41–60	143 (47.4)
	61–79	145 (48.0)
	≥80	2 (0.7)
Educational level	Primary school or below	134 (44.4)
	Secondary school	110 (36.4)
	Postsecondary	35 (11.6)
	University or above	23 (7.6)
Marital status	Single	2 (0.7)
	Married	278 (92.1)
	Divorced/widowed	22 (7.3)
Current residence	Rural	124 (41.1)
	Urban	121 (40.1)
	Urban-rural fringe	57 (18.9)
Occupational status	Employed	46 (15.2)
	Farmer/Worker	163 (54.0)
	Retired	55 (18.2)
	Unemployed	38 (12.6)
Health insurance type	Out-of-pocket	4 (1.3)
	Employee Basic Medical Insurance	96 (31.8)
	Resident Basic Medical Insurance	202 (66.9)
Monthly household income	<2000 CNY	88 (29.1)
	2000–5000 CNY	143 (47.4)
	>5000 CNY	71 (23.5)
Smoking history	Yes	84 (27.8)
	No	218 (72.2)
Alcohol consumption	Yes	93 (30.8)
	No	209 (69.2)
Tumor type	Colon	134 (44.4)
	Rectal	159 (52.6)
	Colorectal	9 (3.0)
Tissue typing	Adenocarcinoma	284 (94.0)
	Mucinous adenocarcinoma	9 (3.0)
	Others	9 (3.0)
Tumor stage	Stage I	2 (0.6)
	Stage II	67 (22.2)
	Stage III	169 (56.0)
	Stage IV	64 (21.2)
Length of diagnosis (mo)	0–6	147 (48.8)
	6–12	69 (22.8)
	12–18	42 (13.9)
	>18	44 (14.5)
Neoadjuvant chemotherapy	Yes	86 (28.5)
	No	216 (71.5)
Cycles of chemotherapy	≤2	145 (48.0)
	3–5	80 (26.5)
	≥6	77 (25.5)
Chemotherapy regimens*	FOLFOX	175 (57.9)
	XELOX	87 (28.8)
	FOLFIRI	19 (6.3)
	Others	21 (7.0)
Radiotherapy	Yes	35 (11.6)
	No	267 (88.4)
Targeted therapy	Yes	115 (38.1)
	No	187 (61.9)
Immunotherapy	Yes	6 (2.0)
	No	296 (98.0)
Distant metastasis	Yes	81 (26.8)
	No	221 (73.2)
Enterostomy	Yes	50 (16.6)
	No	252 (83.4)
Comorbidities†	0	224 (74.2)
	1	61 (20.2)
	≥2	17 (5.6)

* In the chemotherapy regimen, FOLFOX refers to oxaliplatin + fluorouracil + calcium folinate, XELOX refers to oxaliplatin + capecitabine, and FOLFIRI refers to fluorouracil + calcium folinate + irinotecan.

† There are concurrent chronic diseases such as hypertension, diabetes, coronary heart disease, chronic obstructive emphysema, asthma, and kidney disease.

### 3.2. Extraction of symptom occurrence rate, severity, and symptom group of patients with postoperative chemotherapy for CRC

The incidence, severity of symptoms, and the extraction results of symptom clusters in patients undergoing postoperative chemotherapy for CRC are shown in Table 2. As shown in the table, the top 3 symptoms of patients after CRC chemotherapy were poor appetite (85.4%), restless sleep (84.4%), and distress (82.5%). The top 3 symptoms in severity ranking were appetite loss, fatigue, and sleep disturbances. This study included 17 symptoms with an incidence rate of more than 20% into factor analysis, and the KMO value was 0.744, and Bartlett sphericity test χ^2^ = 1355.804 (*P* < .001), indicating that it was suitable for factor analysis. Through principal component analysis, 6 initial factors were extracted. Following the clinical criterion that symptom clusters must contain ≥2 associated symptoms (as individual symptoms cannot form clusters), factor 6, containing only 1 symptom, was excluded, ultimately retaining 5 symptom clusters.

**Table 2:** Incidence of symptoms, severity, and extraction results of symptom clusters in patients undergoing postoperative chemotherapy for colorectal cancer (n = 302).

Variables	Prevalence, n (%)	Severity		Factor loading					
		*M (P*_25_, *P*_75_)	Mean	Factor 1	Factor 2	Factor 3	Factor 4	Factor 5	Factor 6
Vomiting	123 (40.7)	0 (0, 3)	1.53	0.791					
Nausea	171 (56.5)	3 (0, 4)	2.38	0.747					
Pain	165 (54.6)	2 (0, 4)	1.96	0.629					
Fatigue	237 (78.5)	4 (2, 5)	3.27	0.611					
Shortness of breath	107 (35.4)	0 (0, 3)	1.12	0.611					
Distress	249 (82.5)	3 (2, 4)	2.71		0.864				
Sadness	226 (74.8)	2 (0, 3)	2.24		0.797				
Sleep disturbance	255 (84.4)	3 (2, 5)	3.16		0.681				
Difficulty remembering	224 (74.2)	3 (0, 4)	2.70			0.773			
Dry mouth	206 (68.2)	3 (0, 4)	2.60			0.651			
Drowsiness	190 (62.9)	2 (0, 3)	1.85			0.592			
Abdominal distension	111 (36.8)	0 (0, 3)	1.19			0.502			
Poor appetite	258 (85.4)	4 (2, 5)	3.49				0.814		
Dysgeusia	216 (71.5)	3 (0, 4)	2.72				0.793		
Diarrhea	138 (45.7)	0 (0, 4)	1.74					0.801	
Constipation	152 (50.3)	1 (0, 4)	2.12					0.797	
Dysphagia	35 (11.6)	0 (0, 0)	0.29	–	–	–	–	–	–
Numbness	223 (73.8)	4 (0, 5)	3.14						0.919
Eigenvalue	–	–	–	24.55	10.07	8.74	8.00	7.07	6.09
Cumulative variance contribution rate (%)	–	–	–	15.83	27.68	38.88	49.64	58.13	64.53

**Figure 1.**
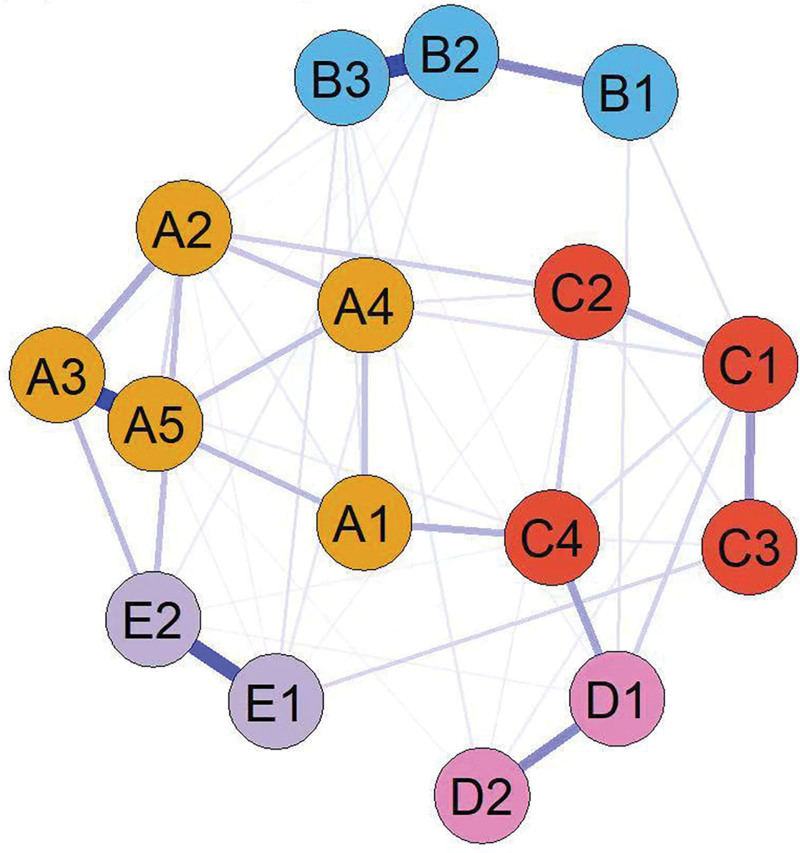
Network structure model of colorectal cancer patients receiving chemotherapy.

According to the characteristics of the symptoms, the identified symptom clusters were categorized and named as follows: chemotherapy-related symptom clusters, psychological symptom clusters, neurotoxicity-related symptom clusters, gastrointestinal-related symptom clusters, and CRC-specific symptom clusters (Fig. 1, symptom network diagram). Different colors indicate the 5 final retained symptom clusters: the chemotherapy-related cluster includes nausea, vomiting, fatigue, shortness of breath, and pain; the psychological cluster comprises distress, sadness, and sleep disturbances; the neurotoxicity-related cluster consists of amnesia, dry mouth, drowsiness, and abdominal distension; the gastrointestinal-related cluster contains appetite loss and altered taste perception; and the CRC-specific cluster features diarrhea and constipation.

### 3.3. Symptom network analysis of patients with postoperative chemotherapy for CRC

**Figure 2.**
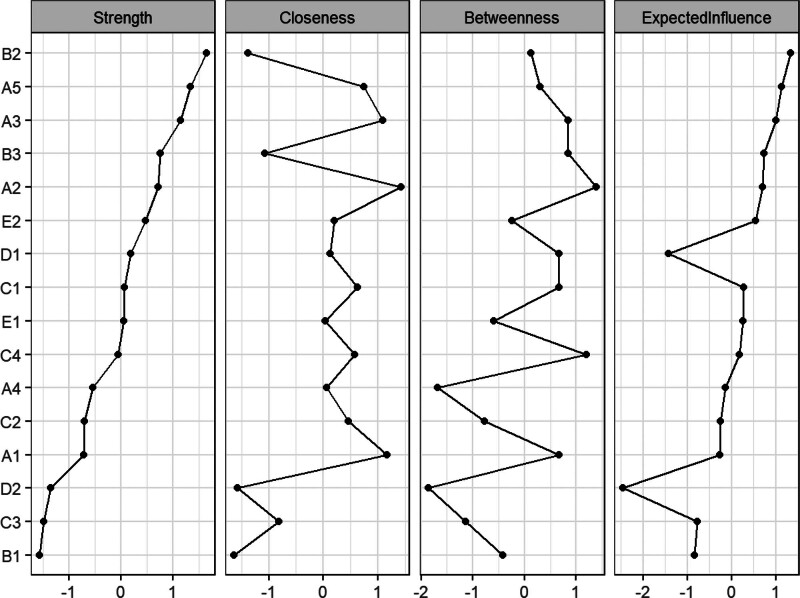
Centrality scores for all variables in the network.

**Figure 3.**
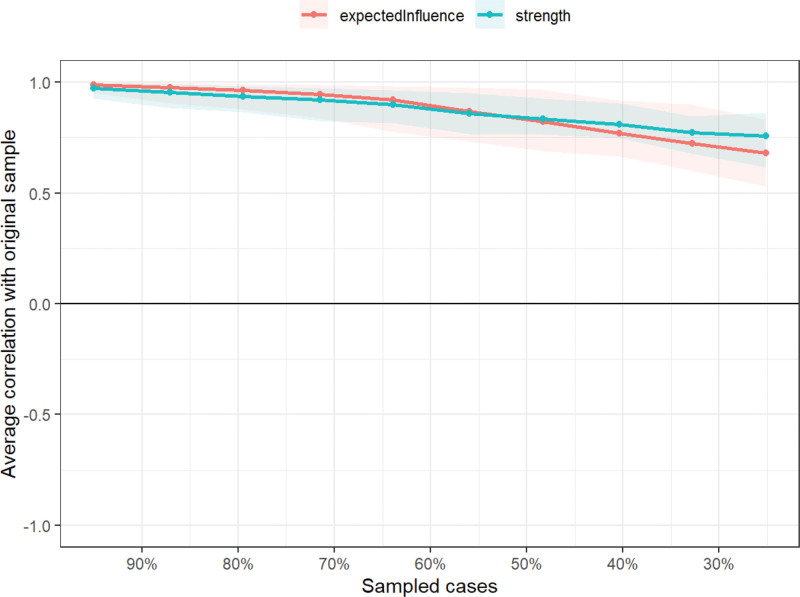
Stability analysis of centrality indicators.

**Figure 4.**
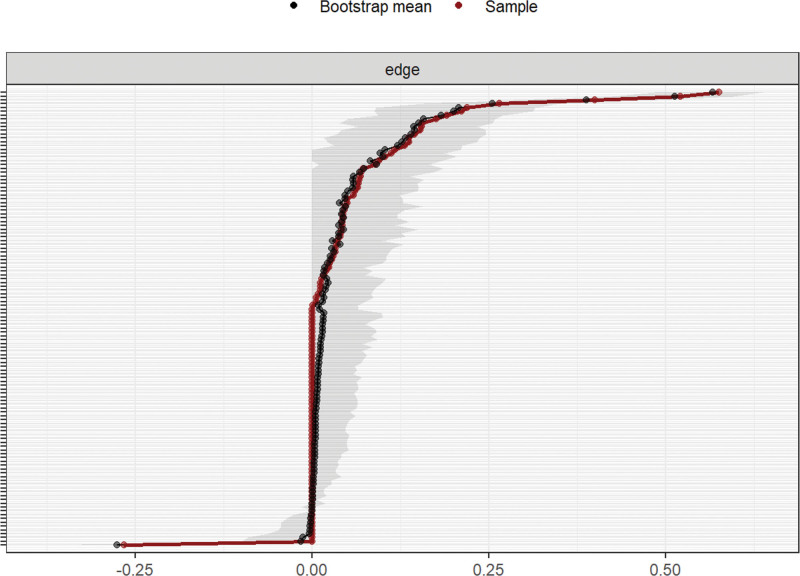
Accuracy analysis of edge weights.

In the symptom network, thicker and darker lines indicate stronger associations between symptoms. For example, there is a positive correlation between “sadness” and “distress,” “poor appetite” and “changes in taste,” and “nausea” and “vomiting,” suggesting that these symptoms often co-occur and may reinforce each other, as shown in Figure 1. As shown in Figure 2, the top 3 symptoms in terms of network node centrality strength and expected influence are distress (robust strength [*r*_s_] = 1.047, robust expected influence [*r*_e_] = 1.047), vomiting (*r*_s_ = 0.983, *r*_e_ = 0.983), and nausea (*r*_s_ = 0.946, *r*_e_ = 0.946). Notably, distress demonstrated the highest node strength and expected influence within the symptom network, identifying it as a central hub that exerts the strongest direct impact on other symptoms. Its severity may ultimately determine the progression of the entire symptom cluster. Furthermore, fatigue has the highest closeness (*r*_c_ = 0.0047), indicating that although it does not have the strongest direct connection, it can indirectly affect other symptoms in the network through the fewest paths and has the potential for rapid spread. As shown in Figure 3, the correlation stability coefficient of network strength is 0.672 and the expected impact is 0.517. Both the correlation stability coefficient of network strength and expected impact are >0.5, indicating that the network stability is good. As shown in Figure 4, the 95% confidence interval of edge weight is small, indicating high network accuracy.

## 4. Discussion

### 4.1. Characteristic analysis of symptom clusters in patients with postoperative chemotherapy for CRC

In this study, 5 symptom clusters were extracted by principal component analysis: chemotherapy-related symptom cluster, psychological symptom cluster, neurotoxicity-related symptom cluster, gastrointestinal symptom cluster, and CRC-specific symptom cluster. Vomiting, nausea, pain, fatigue, and shortness of breath are common side effects of chemotherapy drugs, so factor 1 was named the chemotherapy-related symptom cluster. Continuous high-stress events reduce patients’ psychological coping ability and make them prone to negative emotions such as distress and sadness. Especially for postoperative stoma patients, the change of defecation habits has a great impact on the patients’ body and mind. Patients generally have moderate to high levels of disease shame and sleep disorders,^[21]^ so factor 2 was named the psycho-symptom cluster. Chemotherapy drugs are generally cytotoxic drugs. While killing and inhibiting tumor cells, they also damage normal cells of the body to varying degrees, resulting in neurotoxic symptoms such as amnesia, dry mouth, drowsiness, and abdominal distension. Therefore, factor 3 was named the neurotoxicity-related symptom cluster. Chemotherapy can prevent cancer cell metastasis, but its associated gastrointestinal side effects, combined with the inability of some postoperative stoma patients to voluntarily control bowel movements, often lead them to restrict food intake in an attempt to manage excretion volume and minimize stoma-related discomfort. These factors may result in poor appetite and even altered taste perception, thereby affecting eating habits. For these reasons, factor 4 was designated as the gastrointestinal-related symptom cluster. CRC surgery can cause intestinal mucosal damage, which reduces nutrient absorption and often leads to postoperative diarrhea. Chemotherapy drugs can also cause intestinal mucosal cell damage, leading to dysfunction or disorder of intestinal mucosal cells, which can cause diarrhea and constipation. Therefore, this study named factor 5 the CRC-specific symptom cluster. This study and Li et al^[22]^ identified 5 symptom groups, but the classification of symptoms is not completely consistent, which may be related to the multifactor regulation of chemotherapy patients’ symptom experience, such as disease characteristics, treatment side effects, treatment methods, etc, which will affect patients’ symptom experience and show different symptom clusters.^[23,24]^

### 4.2. Analysis of symptoms in patients with postoperative chemotherapy for CRC

Advancements in data analysis technology have opened new avenues for understanding patient symptom networks, with the identification of key symptoms within these networks emerging as a major research focus in network analysis. Recognizing critical symptoms within such networks enables targeted interventions. This study employs network analysis to identify distress and fatigue as core symptoms in the symptom profile of postoperative chemotherapy patients undergoing CRC treatment.

#### 4.2.1. Distress is the core symptom of the chemotherapy-related symptom group in patients with CRC after postoperative chemotherapy

This study shows that the node strength of distress symptoms is the highest (*r*_s_ = 1.047, *r*_e_ = 1.047), and it occupies a central position in the symptom network of patients with post-CRC chemotherapy, belonging to core symptoms, which is consistent with the results of Zheng et al.^[9]^ Distress is the most obvious symptom in chemotherapy patients after CRC surgery. Facing the great pain of long-term chemotherapy, patients are prone to negative emotions such as anxiety and sadness. With the accumulation of negative emotions, it may even lead to psychological problems such as anxiety and depression. Studies have shown that unmanaged negative emotions can further aggravate the symptom burden of patients, and adverse emotions can indirectly affect their subsequent treatment and quality of life by affecting their sleep and diet.^[25]^ Therefore, healthcare professionals should promptly monitor and assess patients’ mental health status, implement timely psychological interventions based on individual circumstances to alleviate distressing emotions, and enhance their daily living quality.

#### 4.2.2. Fatigue is the core symptom of psychosomatic symptoms in patients with postoperative chemotherapy for CRC

Symptom network analysis showed that fatigue was the core symptom of psychosomatic symptoms in postoperative chemotherapy patients with CRC, and the occurrence rate of fatigue in the whole network was 78.5%, which was consistent with the results of Rha et al.^[26]^ Fatigue may serve as a critical indicator of postoperative chemotherapy-related symptoms in CRC patients. Its occurrence is associated with multiple factors. First, the tumor itself and its treatment-related side effects – such as mitochondrial dysfunction and inflammatory cytokine release (e.g., interleukin-6 and tumor necrosis factor α) induced by chemotherapeutic agents such as oxaliplatin and 5-fluorouracil – may be key contributors to fatigue development.^[27,28]^ Second, other treatment-associated symptoms, including pain, sleep disturbances (drowsiness, daytime drowsiness, and restless sleep), emotional distress, appetite loss, and nutritional deficiencies, can exacerbate fatigue,^[29–31]^ significantly impacting quality of life. Healthcare providers should promptly assess risk factors and implement appropriate interventions for fatigued patients. Research^[32]^ demonstrates that both aerobic and resistance training effectively alleviate fatigue symptoms. Additionally, exercise therapy and psychosocial interventions have been recommended for fatigue management.^[33]^ Therefore, when patients experience fatigue, clinicians should guide them in personalized exercise regimens (e.g., 3 moderate-intensity aerobic sessions combined with resistance training weekly) while integrating psychosocial interventions such as cognitive behavioral therapy and mindfulness practice. These approaches improve mitochondrial function, suppress pro-inflammatory cytokine release, and correct negative cognitive patterns, thereby achieving synergistic effects of reduced fatigue scores and enhanced symptom relief.^[32,34]^

#### 4.2.3. Poor appetite is the most common symptom in patients with postoperative chemotherapy for CRC

The results of this study showed that poor appetite was the most common symptom in CRC patients during postoperative chemotherapy, with an incidence rate of 85.4% in the whole network. The mechanism of poor appetite after chemotherapy involved the synergistic effect of chemotherapy drugs on multiple systems. Chemotherapy drugs can directly damage the taste bud cells of the tongue, leading to the downregulation of taste receptor function and causing taste disorders, which are often manifested as a metallic taste, increased bitter taste, or aversion to specific foods such as meat. These abnormal taste experiences directly inhibit the appetite of patients.^[35]^ In addition, chemotherapy drugs often directly stimulate the gastrointestinal mucosa, causing nausea, vomiting, or a feeling of fullness, which physically reduces the desire to eat. Second, psychological distress such as anxiety and depression caused by the disease will further aggravate the poor appetite, forming a vicious cycle of physiology and psychology. Sustained inadequate intake can easily lead to malnutrition, weight loss, and sarcopenia, which not only affects the quality of life and physical state of patients but may also weaken their tolerance to chemotherapy, leading to reduced chemotherapy dose or treatment interruption and ultimately affecting the overall efficacy of antitumor therapy. Therefore, identifying and managing poor appetite – a frequent and burdensome symptom – holds significant clinical importance. It should be regarded as one of the core targets in symptom management for postoperative chemotherapy patients with CRC. Early and proactive interventions (such as nutritional support, medication adjustment, and dietary guidance) are crucial for breaking the vicious cycle, maintaining treatment intensity, and improving patient outcomes.

## 5. Limitations

This study has certain limitations. First, the cross-sectional design cannot elucidate the dynamic evolution of symptom networks or establish causal relationships between symptoms. Furthermore, data collection was not restricted to a fixed chemotherapy cycle. Since symptom experience fluctuates with treatment progression, such variation may affect the stability of the constructed symptom network – an inherent challenge in real-world research aimed at achieving broader representativeness. Second, all samples were obtained from only 2 hospitals in a single city, and the limited sample size may compromise the generalizability and stability of the findings. Future studies should adopt multicenter, large-sample, longitudinal designs to repeatedly assess symptom networks at specific time points (e.g., after each chemotherapy cycle), in order to delineate their dynamic trajectories throughout the treatment course and to compare network differences across clinical subgroups (e.g., chemotherapy regimen, disease stage). Such efforts will provide evidence for truly precise symptom management.

## 6. Conclusion

This study, through symptom network analysis, has for the first time identified distress and fatigue as the core symptoms within the symptom network of CRC patients after postoperative chemotherapy, while exploratory factor analysis identified 5 major symptom clusters: chemotherapy-related, psychological, neurotoxicity-related, gastrointestinal-related, and CRC-specific clusters. It is noteworthy that the symptom with the highest prevalence (poor appetite) differed from the symptoms with the highest centrality in the network analysis. This finding precisely highlights the unique value of different analytical approaches: prevalence analysis identifies common symptoms that require universal screening and routine management, whereas network analysis reveals potential key intervention targets within the symptom system; interventions targeting core symptoms (distress, fatigue) may exert a “leveraged effect” on alleviating the entire symptom network. Therefore, healthcare professionals should consider distress, fatigue, and poor appetite as simultaneous intervention priorities, albeit with distinct strategies: implementing universal screening and basic management for “poor appetite,” while developing targeted precision intervention strategies (such as cognitive behavioral therapy, activity management) for “distress” and “fatigue.” This approach aims to enhance overall intervention efficiency and patient quality of life.

## Acknowledgments

The authors thank all the patients and staff who participated in this study.

## Author contributions

**Conceptualization:** Bingbing Xiao, Hongyan Kou.

**Data curation:** Bingbing Xiao, Wendan Jing, Tingting Tan.

**Formal analysis:** Bingbing Xiao.

**Funding acquisition:** Hongyan Kou.

**Investigation:** Bingbing Xiao, Wendan Jing, Qijun Lv.

**Methodology:** Bingbing Xiao, Jiayi Wan.

**Software:** Bingbing Xiao, Qijun Lv.

**Supervision:** Jiayi Wan, Jing Zhao.

**Validation:** Jing Zhao, Tingting Tan.

**Visualization:** Bingbing Xiao.

**Writing – original draft:** Bingbing Xiao.

**Writing – review & editing:** Bingbing Xiao.
